# Molecular Aspects of HTLV-1 Entry: Functional Domains of the HTLV-1 Surface Subunit (SU) and Their Relationships to the Entry Receptors

**DOI:** 10.3390/v3060794

**Published:** 2011-06-15

**Authors:** Kathryn S. Jones, Sophie Lambert, Manuella Bouttier, Laurence Bénit, Frank W. Ruscetti, Olivier Hermine, Claudine Pique

**Affiliations:** 1 Basic Science Program, SAIC-Frederick, Inc., NCI-Frederick, Maryland, MD 21702, USA; E-Mail: joneska@mail.nih.gov; 2 INSERM, U1016, Institut Cochin, 22 rue Mechain, 75014, Paris, France; E-Mails: s.lambert@inserm.fr (S.L.); manuella.bouttier@igmm.cnrs.fr (M.B.); laurence.benit@inserm.fr (L.B.); 3 CNRS, UMR8104, 75014, Paris, France; 4 Université Paris Descartes, 75006, Paris, France; 5 Cancer and Inflammation Program, NCI-Frederick, Frederick, Maryland, MD 21702, USA; E-Mail: ruscettf@mail.nih.gov; 6 Service d’Hématologie Adulte, Hôpital Necker, 75743, Paris CEDEX 15, France; E-Mail: ohermine@gmail.com

**Keywords:** retrovirus, viral entry, Env, proteoglycans, neuropilin, GLUT

## Abstract

The initial step in retroviral infection involves specific interactions between viral envelope proteins (Env) and specific receptors on the surface of target cells. For many years, little was known about the entry receptors for HTLV-1. During this time, however, functional domains of the HTLV-1 Env were identified by analyzing the effects of neutralizing antibodies and specific mutations in Env on HTLV-1 infectivity. More recent studies have revealed that HTLV-1 infectivity involves interactions with three different molecules: heparan sulfate proteoglycans (HSPG), the VEGF-165 receptor Neuropilin 1 (NRP-1) and glucose transporter type 1 (GLUT1). Here, we revisit previously published data on the functional domains of Env in regard to the recent knowledge acquired about this multi-receptor complex. We also discuss the similarities and differences between HTLV-1 and other deltaretroviruses in regards to receptor usage.

## 1. Introduction

Like other enveloped viruses, retroviruses enter target cells by fusing their membrane with the membrane of target cells. This process is initiated by interaction of the surface subunit (SU) of the virally-encoded envelope glycoprotein (Env) with host cell receptors. Data from several independent groups have determined that three cell surface proteins are involved in HTLV-1 entry: glucose transporter 1 (GLUT1), neuropilin-1 (NRP-1) and heparan sulfate proteoglycans (HSPG). This knowledge provides a new perspective on HTLV-1 tropism and associated pathologies. This also raises many questions about the molecular events that occur during entry, in particular how the HTLV-1 SU interacts with each of the receptor molecules. The impact of the receptors on the selectivity and modulation of HTLV-1 infection *in vivo* has been recently reviewed in detail [1,2]. In this review, we reexamine what is known from earlier studies about the functional domains of Env in light of the recent insight into the receptor complex. We will also present recent data obtained with the other members of the HTLV family and discuss their implications in terms of receptor usage.

## 2. The Host Cell Actors: The HTLV-1 Receptors

Here, we will only briefly summarize the evidence that identified the HTLV-1 receptor molecules and led to the proposal of a multi-receptor model for HTLV-1 entry.

### 2.1. Identification of the Roles of GLUT1, NRP-1 and HSPGs in HTLV-1 Entry

The identification of GLUT1 started with the observation that expression of a fragment of the HTLV-1 SU in cells prevents medium acidification [3]. Since it is known for other retroviruses that SU interacts with their receptors when coexpressed in cells, the authors hypothesized that the HTLV-1 receptor might be related to proton-dependent lactate production. Investigation of different members of the glucose transporter family led to the observation that one of these, GLUT1, was indeed able to bind the SU and promote HTLV-1-Env mediated particle entry. The same study showed that the residues D106 and Y114 of the SU were involved in GLUT1 binding [3]. A subsequent study from another group demonstrated that GLUT1 is required for HTLV-1 infection of CD4^+^ T cells [4].

In parallel, the role in HTLV-1 entry of another protein, Neuropilin 1 (NRP-1), was investigated. NRP-1 is a cell surface protein known to function as a co-receptor for certain heparin-binding pro-angiogenic cytokines, principally members of the vascular endothelial growth factor (VEGF) family, and for class 3 semaphorins (reviewed in [5]). It was noticed that a number of features of NRP-1 paralleled characteristics of the HTLV-1 receptor, including a high degree of conservation among vertebrate species [6], the absence of a homolog in the *Drosophila* genome, overexpression in transformed cells [7] and upregulation upon T-cell activation [8]. It was subsequently demonstrated that NRP-1 binds HTLV-1 SU and is required for efficient HTLV-1 entry. The same study also showed that NRP-1, GLUT1 and the HTLV-1 SU form a stable tripartite complex when coexpressed in cells [9].

The role of the third player of HTLV-1 entry, HSPGs, was the first of the three molecules identified to be important for HTLV-1 entry, through experiments showing that removal of HSPGs from cell surface abolished binding of the HTLV-1 SU as well as HTLV-1-Env mediated infection of target cells [10]. Later studies showed that HSPGs were also required for efficient HTLV-1 entry into primary T cells and dendritic cells [11,12]. The region of the SU involved in HSPG binding was characterized using the fact that, unlike HTLV-1, binding of the HTLV-2 SU to target cells does not depend on HSPGs. Analysis of various HTLV-1/HTLV-2 SU chimera demonstrated that binding to HSPGs involved the C-terminal domain of the HTLV-1 SU (amino-acids 215–313) [13].

### 2.2. Cooperation between the HTLV-1 Receptors

The fact that NRP-1 and GLUT1 can form a complex in the presence of HTLV-1 Env suggested that these two molecules work together to promote HTLV-1 entry. Such cooperation was also recently functionally demonstrated by data showing that inhibition of HTLV-1 entry into primary astrocytes required the blocking of the interactions with both NRP-1 and GLUT1 [14].

**Figure 1.**
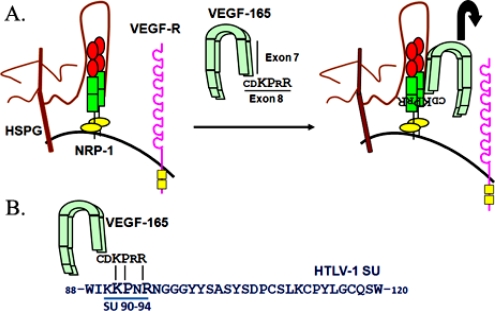
(**A**) Schematic representation of the binding of VEGF-165 to heparan sulfate proteoglycans (HSPG)/neuropilin-1 (NRP-1) complexes prior to interaction with the VEGF receptor. The position in VEGF-165 of the exon 7-encoded sequence, which binds to HSPGs, is indicated. The primary amino acid sequence of the exon 8-encoded sequence, which directly binds to the b domain of NRP-1, is also shown. The a, b and c domains of NRP-1 are painted in red, green or yellow, respectively. (**B**) Homology between the exon 8-encoded domain of VEGF-165 and the amino acid 90–94 region of the HTLV-1 surface subunit (SU).

Previously, HSPGs and NRP-1 have been shown to cooperate while functioning as co-receptors for the pro-angiogenic factor VEGF-165. Initial binding to cells is believed to involve interactions of VEGF-165 with both NRP-1 and heparin sulfate (HS) chains, followed by interaction of VEGF-165 with its signaling receptor VEGF-R [15]. Structural and functional studies indicate that VEGF-165 directly binds to both NRP-1 and heparin, and that NRP-1 and heparin also directly bind to one another, allowing VEGF-165 dimerization and stable binding on cells [16]. The regions of VEGF-165 responsible for binding heparin and NRP-1 map to exon 7 and exon 8, respectively, with three residues in exon 8 (KPxR) critical for direct NRP-1 binding to VEGF-165 [16] (Figure 1).

**Figure 2.**
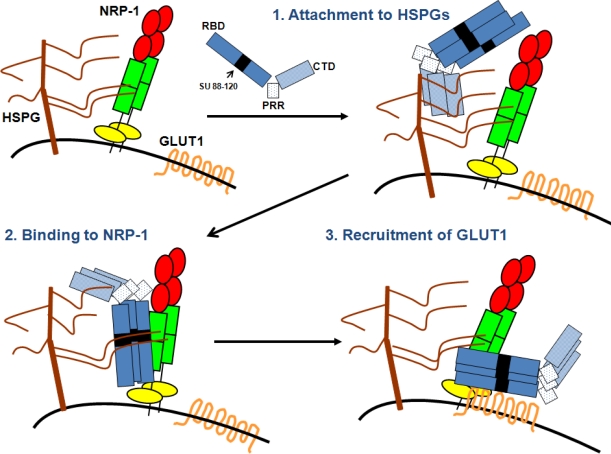
A multireceptor model for HTLV-1 entry. HSPGs, NRP-1 and GLUT1 expressed on the surface of target cells work together to promote HTLV-1 entry. Step 1: The HTLV-1 SU interacts with HSPGs via its C-terminal domain (CTD), which allows the initial attachment and concentration of HTLV-1 particles at the cell surface, Step 2: HSPGs interaction with both the SU and NRP-1 as well as direct binding of the SU to the b domain of NRP-1 via the 90–94 domain allow the recruitment of NRP-1; Step 3: The stable binding of the SU to HSPGs/NRP-1 complexes triggers conformational changes within the SU allowing GLUT1 binding, notably via residues D106 and Y114 of the SU. The HTLV-1 SU is represented in blue with its three modules: the Receptor-Binding domain (RBD), the Proline-Rich Region (PRR) and the C-terminal domain (CTD). The black box within the RBD represents the 88–120 region that contains the KPxR motif and the D106/Y114 residues responsible for NRP-1 or GLUT1 binding, respectively.

**Figure 3.**
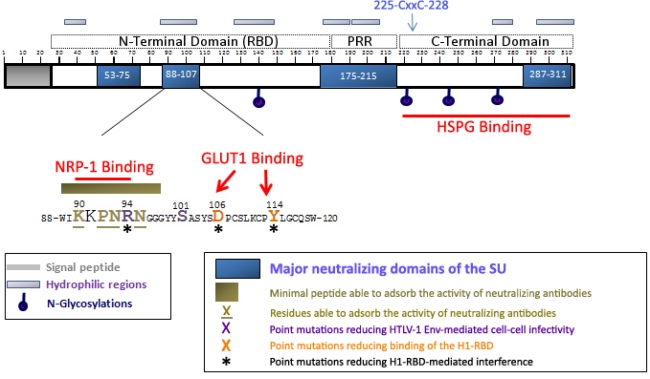
Localization of the neutralizing regions and the domains and residues involved in HSPGs, NRP-1 and GLUT1 binding within the HTLV-1 SU. The 90–94 motif identified as critical for direct NRP-1 binding [17] corresponds to a minimal neutralizing epitope [43], and contains the R94 residue required for HTLV-1 particle infectivity [44]. R94, as well as D106 and Y114 that mediate binding of the H1-RBD to target cells [3] are required for H1-RBD-mediated receptor interference [36]. The C-terminal domain of the SU contains the determinants for HSPG binding [13].

Recently, it has been shown that HSPG and NRP-1 also work together to promote HTLV-1 Env binding. Indeed, like VEGF-165, HTLV-1 SU binds via a HS-dependent manner to the same domain of NRP-1 important for binding VEGF-165 (NRP-1 b domain, see Figures 1 and 2). Moreover, it was discovered that the same KPxR motif critical for direct binding of VEGF-165 exon 8 to NRP-1 was present in the SU of HTLV-1, in a region previously shown to be important for infectivity (Figures 1B and 3). Further studies revealed that a pentapeptide corresponding to the region of the HTLV-1 SU encoding this motif (aa 90–94, KKPNR) directly binds to NRP-1 *in vitro* and was sufficient to block HTLV-1 entry into primary T or dendritic cells [17]. Thus, the HTLV-1 SU stably binds to NRP-1 through both HSPG-mediated and direct interactions by mimicking the NRP-1 ligand VEGF-165.

### 2.3. A Multireceptor Model for HTLV-1 Entry

The data described above support a multi-receptor model for HTLV-1 entry that involves three phases: virus attachment, virus binding and virus/cell fusion (recently discussed in [2]) (Figure 2). Virus attachment is mediated through interaction of the SU with the HS chains, most likely on cell surface HSPG. Then, because HS chains are able to interact with NRP-1, prior binding of the SU to HSPGs increases the probability of the SU to encounter NRP-1 and allows stable binding to NRP-1, ensuring thereby the transition between virus attachment and virus binding. This binding of the SU to HSPG/NRP-1 complexes allows exposure of the GLUT1-binding domain, presumably via conformational changes. Finally, the SU binds to GLUT1, triggering the fusion process that allows entry of the HTLV-1 core in the cell cytoplasm.

The notion that HSPG/NRP-1 are important for the initial binding step is supported by previous observations that HTLV-1 SU and particle binding are dramatically reduced following removal of HS chains or upon blocking NRP-1 interactions by incubation of target cells with either VEGF-165 exon 7 or exon 8-like peptides [12,17]. Blocking interactions with HS chains and NRP-1 also decreased infection of CD4^+^ T cells by HTLV-1 [11]. Previous studies indicate that at least some of the HS chains are in the form of HSPGs, likely with syndecans as core proteins [13]. However, since NRP-1 itself is modified by HS on certain cell types [18], it leaves two possibilities for SU binding; the SU binds to HSPGs which interact with NRP-1 or the SU interacts directly with HS conjugated to NRP-1. A role of GLUT1 in the fusion step subsequent to the initial binding is supported by the observation that the level of GLUT1 expression on target cells correlates with the titer of HTLV-1 Env-pseudotyped viruses but not with the level of binding of the HTLV-1 viral particle or soluble full length HTLV-1 SU protein [3,13,14,19].

## 3. The Viral Actor: The HTLV-1 SU Protein

### 3.1. Function of Retroviral SU Proteins

Retroviral envelope glycoproteins (Env) are type-I transmembrane proteins synthesized as a precursor that is co-translationally imported into the endoplasmic reticulum, where it subsequently undergoes a number of maturation steps, including folding, oligomerization and N-glycosylations. Upon completion of these processes, the Env precursor passes through the Golgi apparatus to the trans golgi network, where it is cleaved by a cellular protease of the furin family into SU (Surface) and TM (Transmembrane) subunits [20]. SU-TM complexes organized as trimers are then transported to the surface of infected cells, where incorporation into the budding particles occurs [21].

Retrovirus entry occurs by fusion of the viral envelope either with the cell plasma membrane [22], or with endosomal membranes following internalization via a endocytic pathway [23–25]. In both cases, the entry process *per se* occurs through an initial binding step mediated by the SU and a subsequent fusion step mediated by the TM. Accordingly, the SU contains the determinants for receptor binding and the TM has the hydrophobic fusogenic peptide in its N-terminal region. During the intracellular journey of SU/TM complexes and prior to contact with the receptor, the TM is maintained by close association with the SU in a fusogenic-inactive metastable conformation in which the fusion peptide is buried. This prevents premature membrane fusion that could lead to Env inactivation and cell toxicity. The key event for TM activation is alteration of the SU/TM interactions, which allows the TM to acquire its fusogenic state. In the case of murine leukemia virus (MLV) Env, SU/TM dissociation is triggered upon binding of the SU to its receptor mCAT-1 and relies on the disruption of an intersubunit disulfite bond between cysteines in a CxxC motif in the SU and a CX_6_CC motif in the TM [26,27]. For human immunodeficiency virus type 1 (HIV-1) Env, TM activation requires successive contacts with two entry receptors, the primary binding receptor CD4 and the co-receptor (CXCR4 or CCR5), with the latter contact triggering SU/TM dissociation [28]. The conformational changes required for chemokine binding are also facilitated by thiol/disulfide rearrangement in the SU-gp120, which involves gp120 binding to cell surface protein disulfite isomerase [29].

For all retroviruses, final activation of the fusion process occurs through successive refolding events of the TM resulting in the projection of the N-terminal fusion peptide. This involves the formation of a six-helix coiled-coil bundle that brings the viral and target membranes in close proximity and triggers membrane fusion. Since this review is focused on the interactions between the SU subunit and the cell receptors, details of these processes will not be described here but can be found in recent reviews [22,30].

### 3.2. General Organization of the HTLV-1 SU

The HTLV-1 *env* gene encodes a 488 amino acid precursor protein, which generates a 62 kDa protein (gp62) after addition of five N-glycan chains, four of which are in the SU (Figure 3) [31]. The gp62 is cleaved at a trypsin-like proteolytic site spanning residues 309–312 into the mature SU (gp46) and TM (gp21) subunits [31]. The SU is entirely extracellular and remains linked to the virus through binding to the TM, which is embedded in the viral envelope.

No direct structural data are available for the HTLV-1 SU. However, structural domains have been predicted based on sequence homology with SU proteins of gammaretroviruses. Structural and functional studies of MLV Env revealed that the SU is organized into three structural modules: an N-terminal receptor binding domain (RBD) and a C-terminal domain (CTD) separated by a short proline-rich region (PRR) [32–34]. Alignment of the amino acid sequences of the HTLV-1 and Friend-MLV SU predicted that the HTLV-1 SU is similarly organized, beginning after the signal peptide (residues 1–25), with an N-terminal region (26–180), a Proline-Rich Region (181–215) and a C-terminal domain (216–312) [35,36] (Figure 3). Analysis of MLV/SU chimeras demonstrated that the N-terminal domain of the HTLV-1 SU can complement the MLV RBD and confer HTLV-1 tropism to the resulting protein, indicating that the HTLV-1 residues important for receptor interactions map within the predicted HTLV-1 RBD [36]. Later studies with a soluble form of the HTLV-1 RBD revealed that this domain is sufficient for binding to the cell surface of target cells [36]. However, binding experiments performed with the full length HTLV-1 SU further demonstrated that regions outside the RBD contain important determinants for SU binding to target cells [13]. Residues outside the RBD have also been shown to encode viral entry determinants for several different gammaretroviruses [37,38].

In gammaretroviruses, the PRR domain is believed to represent a hinge region that facilitates the conformational changes induced by receptor binding [32]. The HTLV-1 SU PRR region has been shown to complement the homologous domain of MLV SU [35], suggesting that it plays a similar role in HTLV-1 SU refolding. The CTD of HTLV-1 SU contains a typical disulfide isomerization motif (CxxC; residues 225 to 228, see Figure 3) homologous to the motif in MLV important for conformational changes required for fusion that occur after receptor binding. Mutation of C225 blocks HTLV-1 Env-mediated infection and cell-cell fusion, but dithiothreitol reverses this effect. This indicates that the thiol of this residue is responsible for the disulfite isomerization that occurs following receptor binding, thus disrupting the SU-TM bond and allowing fusion mediated by the TM [39]. The Ser residue at position 101 also controls SU/TM association [40] (Figure 3). Details of the mechanism of HTLV-1 gp41-mediated fusion have been described elsewhere [41,42].

### 3.3. Functional Residues of the HTLV-1 SU

One approach to identifying functional domains of retroviral envelope proteins, notably the receptor binding determinants, is to identify the epitopes of neutralizing antibodies which block Env-mediated functions. In the case of HTLV-1, analysis of the specificities of anti-HTLV-1 neutralizing antibodies identified four functional regions in the SU: two N-terminal regions located between residues 53–75 and 86–107 [43,45], a central region located between residues 175-199 [46,47] and a C-terminal one located between residues 287–311 [45] (also reviewed in [48]) (Figure 3).

The 86–107 region was initially mapped from antibodies raised against HTLV-1 peptides corresponding to regions of the SU predicted to be hydrophilic. Adsorption of the neutralizing activities using a set of shorter peptides identified six residues (KKPNRN) at position 90 to 95 as the minimal neutralizing epitope in this domain [43] (Figure 3). Previous analysis of mutants carrying single amino acid changes in HTLV-1 SU identified two residues in this region critical for cell-cell transmission of the virus: one (R94) which maps in the minimal neutralizing epitope and another in the larger (S101) [44]. The central 175–199 region overlaps with the PRR, which has been shown for gammaretroviruses to mediate the SU refolding events that occur following binding to the receptor, resulting in activation of fusion by the TM. The observation that the MLV TM can become fusogenic when present on a chimeric molecule with the HTLV RBD and PRR indicates that the HTLV PRR also functions to transmit the signal from the RBD to the CTD [36].

### 3.4. Relationships between the Neutralizing Domains of the SU and the Residues Involved in Receptor Binding

Of the four neutralizing regions of HTLV-1 SU described above, the two N-terminal regions spanning residues 53–75 and 86–107 are located within the predicted HTLV-1 RBD. The second of these contains residues that were recently shown to be involved in interactions with NRP-1 and GLUT1 (Figures 1 and 3). Located within the minimal neutralizing SU 90–98 epitope are residues that, as described above, mediate direct binding of the SU to NRP-1: a pentapeptide corresponding to residues 90–94 was shown to block both the binding to, and infection of, cells by HTLV-1, and to be capable of directly binding to NRP-1 [17].The arginine residue within this motif is the same residue (R94) shown in early studies to be important for cell-cell transmission of the virus [44]. The 86–107 region also encodes one of the two residues shown to be involved in GLUT1 binding (D106); the other residue, Y114, is very close to this region [3]. The importance of these three residues for receptor interactions is also supported by observations that expression of an SU fragment corresponding to the H1-RBD (aa 1–215) in target cells reduced the titer of HTLV-1 Env-pseudotyped viruses, presumably by receptor interference, but that H1-RBD carrying a mutation in R94, D106, or Y114 did not [36].

HTLV-1 is a member of the deltaretrovirus family that includes the closely related primate T-lymphotropic viruses (PTLVs, see below) and the more distantly related bovine leukemia virus (BLV). A structural model for the SU of bovine leukemia virus, which shares 36% identity in amino acid sequence with the HTLV-1 SU, was previously generated. This model predicted that the aa 97–106 region of the BLV SU, which is the homologous to the aa 92–103 region of the HTLV-1 SU, is exposed at the surface of the globular structure of the protein [49]. The BLV SU 97–106 region was also described earlier as a neutralizing epitope [50]. However, the KPxR motif involved in NRP-1 binding, which is conserved in all the PTLV SU sequences (see below), is not found in this region of BLV, in agreement with early reports that HTLV-1 and BLV use different receptors [51].

The C-terminal neutralizing region, aa 287–311, also includes a region previously reported to interact with a component of the proposed HTLV-1 receptor complex. Studies with chimera generated between the HTLV-1 SU and the SU of HTLV-2, which does not efficiently bind HSPG, indicate that the residues involved in binding HSPGs map to the CTD (aa 215–313). It would be of interest to investigate whether deletion in the 287–311 domain reduces HSPG binding, which would explain the neutralizing activity of antibodies directed to this part of the SU.

### 3.5. Conformational Changes in the HTLV-1 SU

As mentioned above, attachment to HSPGs is mediated by the C-terminal region of the SU while NRP-1 and GLUT1 binding occur via the N-terminal region. This distance between the HSPGs and NRP-1/GLUT1 interacting domains are compatible with successive binding events. In contrast, the interacting domains for NRP-1 (aa 90–94) or GLUT1 (D106, Y114) are only separated by 16 residues (Figure 3). This is consistent with the model we proposed, in which the stable SU binding to HSPG/NRP-1 complexes triggers conformational changes allowing the subsequent recruitment of GLUT1. In this line, it has been reported that the level of cell surface GLUT1 does not correlate with the level of binding of the HTLV-1 viral particles or with full-length HTLV-1 SU [13,19], but does correlate with the level of binding of the N-terminal H1-RBD fragment (aa 25–215) [3]. This suggests that the isolated H1-RBD (N-terminal domain) may have conformational flexibility which allows it to bind to GLUT1 independently of conformational changes triggered in the full length SU by NRP-1 binding (Figure 3). Since SU are organized as trimers, NRP-1 and GLUT1 could also simultaneously bind to distinct monomer within SU trimers, as reported for the HIV-1 SU [52]. This could explain the fact that tripartite SU/NRP-1/GLUT1 complexes can be detected in cells [9].

## 4. Comparison between HTLV-1 and the other PTLVs

HTLV-1 is one of a group of deltaretroviruses that infect old world primates and humans, referred to as primate T-lymphotropic viruses (PTLVs). The viruses in this group that infect humans (HTLVs) are believed to have originated from interspecies transmission of simian T-lymphotropic viruses (STLVs). To date, the PTLV group includes three human viruses for which a simian counterpart is known (HTLV-1/ STLV-1, HTLV-2/ STLV-2, and HTLV-3/ STLV-3) as well as two viruses (HTLV-4 and STLV-5) for which no counterpart has yet been identified [53–55].

### 4.1. Difference between HTLV-1 and HTLV-2

**Figure 4.**
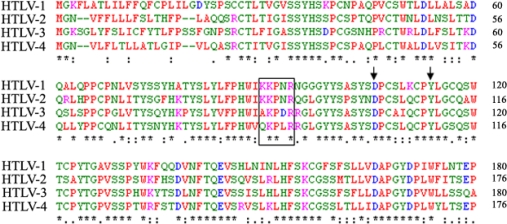
Alignment of the amino acid sequence of the HTLV-1 RBD with the corresponding regions of the HTLV-2, HTLV-3 and HTLV-4 SU. The KKPNR motif of HTLV-1 that mediates direct binding to NRP-1 is boxed and the D106 and Y114 residues that mediate binding to GLUT1 are indicated by arrows. The alignment was performed with the Clustal W program using the following accession numbers: HTLV-1: Genbank AAC82582; HTLV-2: GenBank M10060; HTLV-3: GenBank EU649782 and HTVL-4: NCBI NC_011800. An asterisk indicates identical residues, a colon indicates conserved substitutions and a period indicates semiconserved substitutions.

Among the PTLV group, nearly all of the studies performed to date have examined the *in vivo* tropism and receptor usage of HTLV-1 and/or HTLV-2. The nucleotide sequence homology of the genomes of these two viruses is approximately 60% [56] and their SU proteins share 65% identity on an amino acid level [57] (see Figure 4). The notion that these two viruses share the same receptor is based on early observations that target cells first infected with HTLV-1 became resistant to HTLV-2 infection [51]. However, the *in vivo* tropism of HTLV-1 and HTLV-2 is not identical. HTLV-1 is found primarily in CD4^+^ T cells, while HTLV-2 is found primarily in CD8^+^ T cells [58]. Moreover, the ability of these viruses to transform primary T cells *in vitro* parallel the *in vivo* observations: HTLV-1 preferentially transforms CD4^+^ T cells and HTLV-2 preferentially transforms CD8^+^ T cells [58]. Studies using HTLV-1/HTLV-2 recombinant viruses have mapped this difference to the region of the genome encoding Env: HTLV-2 viruses carrying the HTLV-1 Env preferentially transform CD4^+^ cells, while HTLV-1 viruses with HTLV-2 Env preferentially transform CD8^+^ T cells [59]. While this difference could be due to steps in the viral life cycle after entry, one explanation for the distinct T-cell tropism of HTLV-1 and HTLV-2 could be differences in the receptors the viruses use to enter T cells. Both primary CD4^+^ and CD8^+^ T cells up-regulate NRP-1 upon activation [60], consistent with previous reports that HTLV-1 and HTLV-2 bind to activated, but not naïve, T cells [61,62]. Moreover, reducing levels of NRP-1 has been shown to reduce the titer of both HTLV-1 and HTLV-2 Env-pseudotyped viruses [9]. In contrast, major differences in respect to HSPG and GLUT1 usage have been reported. Removal of HSPGs have been shown to dramatically reduce binding of the HTLV-1, but not HTLV-2, full-length SU to target cells [13]. It was also observed that activated primary CD4^+^ or CD8^+^ T lymphocytes have a reciprocal phenotype in regards to the cell surface expression of HSPG and GLUT1, with a high HSPG/low GLUT1 phenotype for activated CD4^+^ T cells and a low HSPG/high GLUT1 for activated CD8^+^ T cells. Moreover, an increase in HTLV-1 but not HTLV-2 particle internalization was observed upon overexpression of HSPG in CD8^+^ T cells while transfection of GLUT1 in a CD4^+^ T cell line only enhanced the internalization of the HTLV-2 particle [13]. Hence, at least in T cells, HTLV-1 and HTLV-2 do not appear to use the exact same receptor complex, with HTLV-2 being more dependent on the level of GLUT1 and HTLV-1 on HSPGs. However, HTLV-1 and HTLV-2 appear to both interact with NRP-1 and GLUT1, explaining why they belong to the same receptor interference group [51].

The three KPxR residues of the HTLV-1 SU that mediate direct binding to NRP-1 (K91, P92 and R94) and the two residues involved in GLUT1 binding (D106 and Y114) are conserved between the HTLV-1 and HTLV-2 SU [17,63] (Figure 4). Moreover, R90 of HTLV-2, which is the equivalent of R94 of HTLV-1, has been shown to be critical for HTLV-2 infectivity [57]. These findings are fully consistent with the notion that HTLV-1 and HTLV-2 both use NRP-1 and GLUT1 during entry.

### 4.2. Receptor Usage of Other PTLV

HTLV-1 and its simian counterpart STLV-1 are highly related viruses which can share up to 98% nucleotide sequence homology [64]. The entire SU sequences of HTLV-1 and STLV-1 are highly conserved and notably, the aforementioned residues K91, P92, D106 and Y114 are identical between a number of HTLV-1 and STLV-1 isolates [65]. This strongly suggests that like HTLV-1, STLV-1 uses HSPG, NRP-1 and GLUT1 as entry receptors. High homology between HTLV-2 and STLV-2 isolates [66] may similarly indicate that the human and simian type 2 also share the same entry mechanism.

The HTLV-3 genome has been sequenced [67] and the primary amino acid sequences of the HTLV-1, HTLV-2 and HTLV-3 Env have been compared [63]. Interestingly, although the KKPNR sequence is not strictly conserved in the HTLV-3 SU, the three residues in the KPxR motif shown to be critical for binding to NRP-1 are present in HTLV-3. Moreover, the D106 and Y114 residues important for GLUT1 binding are also strictly conserved between the HTLV-1, HTLV-2 and HTLV-3 sequences (Figure 4). The only functional studies with the HTLV-3 Env reported to date revealed that, unlike HTLV-1 and HTLV-2, the HTLV-3 SU can bind to primary resting CD4^+^ T cells that do not express detectable levels of HSPG, NRP-1 or GLUT1 [63]. Blocking interactions with either HSPGs or NRP-1 reduced the level of binding of HTLV-3 SU and the titer of HTLV-3 Env-pseudotyped viruses, but the effect of blocking these interactions was less dramatic than for HTLV-1. It was also reported that, as for HTLV-1, the level of GLUT1 correlates with the titer of HTLV-3 Env-pseudotyped virus but not with the level of binding of the HTLV-3 SU [63]. These finding suggest that as for HTLV-1, HSPG and NRP-1 may participate in the initial binding step and GLUT1 in the final fusion step of the HTLV-3 entry process. However, other molecules expressed by resting T cells appear to be also involved, that presumably can substitute for HSPG and/or NRP-1 during the initial stages of binding.

The nucleotide sequence of the *env* gene is not yet available for STLV-5 but the sequence for one HTLV-4 isolate has been reported (accession NC_011800.1). Examination of this sequence reveals that both the KPxR motif and D106 and Y114 of the HTLV-1 SU are conserved in the amino acid sequence of the HTLV-4 SU (Figure 4), suggesting that HTLV-4 may also use NRP-1 and GLUT1 as entry receptors.

## 5. Conclusions

The functional domains of the HTLV-1 SU were extensively studied long before the identification of the cellular receptors. These earlier studies on Env and recent work on the receptors now converge to identify the N-terminal domain (NRP-1 and GLUT1 binding) and the C-terminal domain (HSPG binding) of the HTLV-1 SU as critical determinants for HTLV-1 entry. Analysis of the primary sequence of other members of the PTLV family predicts that some members may also use NRP-1 and GLUT1 as entry receptors, but may differ from HTLV-1 in HSPG usage. Further studies are clearly needed to better define the receptor complex used by each PTLV member and understand the impact of virus/receptor interactions on the *in vivo* tropism and infection by HTLV-1 and the other PTLVs.
